# Recent advances in intravenous anesthesia and anesthetics

**DOI:** 10.12688/f1000research.13357.1

**Published:** 2018-04-17

**Authors:** Mohamed Mahmoud, Keira P. Mason

**Affiliations:** 1 Department of Anesthesiology, Cincinnati Children’s Hospital Medical Center, University of Cincinnati, 3333 Burnet Avenue, Cincinnati, OH, 45229, USA; 2 Department of Anesthesiology, Critical Care and Pain Medicine, Boston Children’s Hospital and Harvard Medical School, 300 Longwood Avenue, Boston, MA, 02115, USA

**Keywords:** Intravenous Anesthesia, Midazolam, Xenon, Propofol

## Abstract

Anesthesiology, as a field, has made promising advances in the discovery of novel, safe, effective, and efficient methods to deliver care. This review explores refinement in the technology of soft drug development, unique anesthetic delivery systems, and recent drug and device failures.

## Introduction

The first generation of intravenous (IV) agents for anesthesia induction and maintenance, as an alternative to volatile agents, dates back to the introduction of thiopental in the 1930’s. Since then, propofol, ketamine, etomidate, dexmedetomidine, and benzodiazepines represent some of the more significant contributions to IV anesthetic or sedative agents. Researchers continue to develop both new formulations of these existing agents and new chemical entities in strides to improve safety, predictability, efficacy, onset, and recovery profile and to minimize side effects. Novel drug development is challenging, costly (2.5 billion dollars on average), and risky ^1^. Only 1 in 10 drugs in phase I development will go on to obtain Food and Drug Administration (FDA) approval ^2^, and some drugs are withdrawn from the market, even after approval, because of unanticipated limitations and drawbacks.

Since its introduction, target-controlled infusion (TCI) technology has evolved from a research tool into a now-routine part of anesthesia delivery in many countries (USA excluded) worldwide. Advancement in this technology has the potential to expand the precision, reliability, efficacy, and safety of IV anesthesia delivery.

This review of the literature will highlight the recent developments and failures in drug and device innovations and novel drug delivery systems.

## New innovations in drug development

Recent and evolving drug innovations are primarily focused on modifying the chemical structures of existing drugs or drug classes with intent to improve their pharmacodynamic, pharmacokinetic, and side effect properties. The following section reviews remimazolam, etomidate, and propofol derivatives and alternatives which have been formulated to offer improvements on the parent compounds.

### Remimazolam and ADV6209

Midazolam is a widely used sedative and anxiolytic administered as a popular sedation choice and is used for patient care in a variety of inpatient and outpatient settings. Some drawbacks to the use of midazolam include lack of analgesia and prolonged recovery times in patients with liver disease. Remimazolam is a new, short-acting, ester-based anesthetic agent that allows for rapid esterase-mediated metabolism independent of hepatic or renal enzymes and function ^3, 4^. It is currently in phase III clinical development for procedural sedation in the USA. As the name implies, remimazolam combines the properties of two unique drugs already established in anesthesia: midazolam and remifentanil. It acts on GABA receptors, as does midazolam, and exhibits pharmacokinetic properties common to the ester-based opioid remifentanil. The addition of a benzodiazepine to remifentanil targets a hopeful synergy between the two, with improved sedation and anxiolysis. In animal studies, remimazolam produced a more rapid onset and faster recovery than did midazolam ^5^. Remimazolam is primarily cleared by tissue esterase enzymes, carboxylesterases (CESs). CESs participate in the decomposition and metabolism of endogenous and exogenous compounds, which can greatly affect the metabolism of esters, mainly including hCE-1 and hCE-2 ^6^. Accumulation should not occur after infusion of remimazolam because it is primarily cleared by unique tissue esterase enzymes, which convert it into an inactive carboxylic acid metabolite (CNS7054) ^7, 8^. This novel anesthetic agent has a relatively short context-sensitive half-life of 7–8 minutes, even after a 2-hour infusion, and, as with midazolam, can be reversed with flumazenil.

While remimazolam was initially developed for use as a drug for procedural sedation, more studies are currently focused on utilizing this agent for the induction and maintenance of general anesthesia. A recent study examined the feasibility of delivering inhaled remimazolam alone or as an adjunct to remifentanil in rodents. Remimazolam significantly potentiated the analgesic effect of remifentanil, without lung irritation, bronchospasm, or other adverse pulmonary events ^9^.

In the USA, the initial development of remimazolam was intended for sedation for adult colonoscopy ^10– 12^. In the EU, it is undergoing development for general anesthesia in patients undergoing non-cardiac and cardiac surgery, including intensive care unit (ICU) sedation for up to 24 hours after the operation. In Japan, a clinical phase III program in anesthesia has also been conducted. Remimazolam doses of 0.075 to 0.2 mg/kg showed good effect in a phase I trial for safety and efficacy ^8^. Dosages for bolus or infusion are awaited.

Remimazolam has potentially important clinical applications owing to its rapid offset of action and maintenance of stable hemodynamics. Most of the current trials involved infusions of bolus or duration infusions; thus, additional information is required to determine how metabolism is affected by prolonged infusions or repeat boluses. Additional studies are required to provide more data on diverse patient populations (weight, age range, and comorbidities, including renal and hepatic insufficiency). It has not been studied in pregnant patients or the pediatric population. Comprehensive pharmacokinetic and pharmacodynamic as well as safety studies are still required, and many questions remain to be answered before this drug can enter clinical practice.

### ADV6209: new formulation of oral midazolam

A novel formulation of oral midazolam is currently under investigation, with phase I and II trials now started in both adults and children. This innovative 0.2% aqueous midazolam solution has been formulated by combining a sweetener (sucralose), an aroma (orange aroma), and y-cyclodextrin with a citric acid solution of midazolam ^13^. This formulation also appears to improve the longevity of the oral formulation’s shelf-life (slower degradation) ^14^. Initial studies indicate that the pharmacokinetic and pharmacodynamic parameters are unaltered with a 0.2% midazolam formulation, although there are important advantages of increased palatability. A recent study examined the pharmacokinetic characteristics of the ADV6209 oral formulation in children from 6 months to 18 years old, showing that the measured pharmacokinetic parameters of ADV6029 were close to those reported in the literature with other midazolam formulations, such as extemporaneous oral solutions or syrups ^15^. The future role of this drug may be important in offering anxiolysis and sedation with improved patient acceptance and tolerance.

### Etomidate derivatives

Etomidate is a highly potent hypnotic agent which was introduced into clinical practice in 1972 and gained popularity largely because of its relatively benign cardiovascular and respiratory effects. Its side effects include pain at injection site, myoclonus, and nausea and vomiting. The most serious side effect of prolonged etomidate infusions is increased mortality in the critically ill, which is a consequence of the inhibition of 11β-hydroxylase activity and suppression of adrenocortical steroid synthesis. Subsequently, etomidate is no longer administered as a prolonged infusion and is limited to a single bolus, albeit even limited use is still controversial. Recent drug development goals are to retain etomidate’s highly desirable properties while avoiding any suppression of adrenocortical function ^16^.

**Figure 1.**
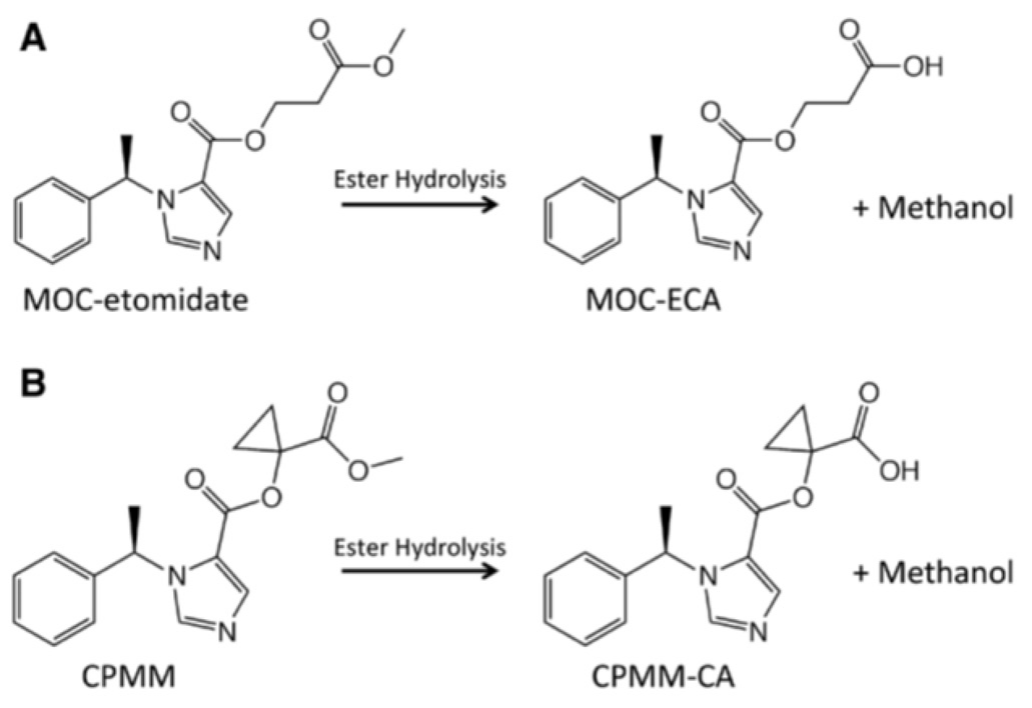
Structures of parent hypnotics and their carboxylic acid metabolites. A) Methoxycarbonyl etomidate (MOC-etomidate) and MOC-etomidate’s carboxylic acid metabolite (MOC-ECA). B) Cyclopropyl-methoxycarbonyl metomidate (CPMM) and CPMM’s carboxylic acid metabolite (CPMM-CA). Permission was obtained for the reproduction of this figure.

Methoxycarbonyl etomidate (MOC-etomidate) ( Figure 1) was the first soft etomidate analogue to be studied. Similar to remifentanil, esmolol, and remimazolam, it contains a metabolically labile ester moiety that is rapidly hydrolyzed by esterases to form a carboxylic acid metabolite and does not produce prolonged adrenocortical suppression in rats after bolus administration ^17^. The resulting carboxylic acid metabolite (MOC-ECA) has GABAA receptor, hypnotic, and adrenocortical inhibitory potencies which are approximately 300-fold lower than those of the parent compound, MOC-etomidate ^18^.

Carboetomidate is an etomidate analogue that does not inhibit steroid production. The reduced ability to suppress *in vitro* and *in vivo* steroid synthesis as compared to etomidate probably reflects carboetomidate’s lower binding affinity to 11β-hydroxylase and its inability to form a coordination bond with the heme iron at the enzyme's active site ^19, 20^. MOC-carboetomidate is meant to combine the rapid metabolism of MOC-etomidate and the minimal adrenal suppression of carboetomidate ^21^. Similar to carboetomidate, MOC-carboetomidate is poorly soluble in water and has a slower onset of action (1.3-minute *in vitro* metabolic half-life) as compared to MOC-etomidate (0.35 minutes).

Cyclopropyl-methoxycarbonyl metomidate (CPMM, also known as ABP-700) ( Figure 1) is a second-generation etomidate. It is a novel, potent, positive allosteric modulator of the GABAA receptor that possesses promising pharmacodynamic and pharmacokinetic profiles in animal studies: it exhibits high potency and hypnosis that reverses within several minutes of stopping continuous infusions of up to 2 hours ^22^. A recent study showed that CPMM (as opposed to MOC-etomidate) infusion is context insensitive because its metabolite fails to reach concentrations in either the blood or the cerebrospinal fluid which are sufficient to have a hypnotic effect ^16^. In August 2017, the Medicines Company announced that it has discontinued the development of MDCO 700 because findings from completed animal studies did not support phase III development of the candidate.

### New propofol derivatives and alternatives

Propofol has been a transformative anesthetic agent since its introduction 40 years ago and is still considered to be a near-ideal anesthetic agent. Its success in the clinical setting is a result of its rapid onset, short duration of action, and minimal side effects. However, propofol is associated with a number of important disadvantages: its oil emulsion adds a risk of bacterial contamination and hyperlipidemia. There may be significant pain upon IV injection and a potentially fatal risk of propofol infusion syndrome (PIS). With these challenges, attention has now shifted to the development of new formulations and alternatives to improve the pharmacologic profile and overcome some of the disadvantages. In this section, we provide an update on the new propofol formulations and alternatives that have been developed to improve on the drawbacks.

***New propofol formulation.*** Currently, there are many anesthetics undergoing clinical development that have been modified to overcome the shortcomings of propofol. However, the ideal drug that is entirely satisfactory does not exist. 2,6-Disubstituted alkylphenols (Haisco HSK3486) are the result of a new propofol modification and seem to possess promising anesthetic properties ^23^. The sedation evaluation of this new formulation demonstrated its better potency as well as faster onset and recovery compared to propofol ^23^.

***Propofol alternatives.*** AZD-3043 (AstraZeneca US, Wilmington, DE, USA) is a water-insoluble drug formulated in an oil emulsion that is similar to propofol. When given IV to rats, AZD-3043 produced rapid-onset hypnosis and rapid recovery within 3 minutes of discontinuing infusions ranging from 20 minutes to 5 hours ^24^. Recent human studies showed that it has fast clearance and a relatively low volume of distribution, consistent with the rapid onset and offset profile ^25, 26^. Metabolism occurs because of the action of plasma and tissue esterase to an inactive metabolite with minimal hypnotic effect. In contrast to propofol, pain with this injection has not been reported ^26^. However, this drug appears to have a number of drawbacks: erythema, chest discomfort, dyspnea, and episodes of involuntary movements ^27, 28^.

Phaxan™ (PHAX, Chemic Labs, Canton, MA) comprises 10 mg/mL alphaxalone and 13% 7-sulfobutylether β-cyclodextrin (betadex) in an aqueous solution. It is a fast onset-offset IV anesthetic like propofol but causes less cardiovascular depression ^29^. Animal studies showed that it has a greater therapeutic index than propofol ^29^. The first human study compared it to an equivalent dose of propofol and demonstrated that, as with propofol, it has fast-onset, short-duration anesthesia and comparatively rapid cognitive recovery but with less cardiovascular depression and airway obstruction. There was no pain on injection ^30^.

***Modifications of propofol emulsion.*** Pain during injection of propofol is not a new problem (incidence 30–90%) ^31^. Some patients recall the induction of anesthesia as the most painful part of the perioperative period ^32^. It has been reported that pain from injection is ranked third among the 33 most common anesthesia problems in outpatient procedures ^33^. The exact mechanism of pain on injection is not known. Factors that appear to influence the incidence and the severity of pain include menstrual cycle, temperature, injection rate, infusion equipment, concentration of propofol, patient age, venous occlusion, and pretreatment medications ^34^.

Modifications to the emulsion formulation have been attempted to overcome pain on injection and infection risk. Microemulsions of propofol are thermodynamically stable and easier to produce; however, their injection causes further pain. Recently, a novel micro to macro (M2M) approach of destabilizing a microemulsion immediately prior to injection was developed ^35^. This novel approach could potentially improve stability and reduce pain on injection.

Another modification of propofol is to increase the proportion of medium-chain triglycerides (MCTs) in the emulsion. MCTs are more polar than long-chain triglycerides (LCTs) and are more rapidly metabolized. Propofol-Lipuro® is a mixed MCT–LCT propofol formulation that has an oil phase that allows a larger proportion of propofol to be dissolved in it and, thereby, reduces pain ^36^. The emulsion did not affect the pharmacokinetics or pharmacodynamics of propofol, caused less pain upon injection, and increased the speed of triglyceride elimination ^37^. This agent has been available outside of the USA since 1999.

### Xenon

Xenon was first administered to humans in 1951 ^38^. It offers the advantages of having the lowest blood gas partition coefficient of any anesthetic, being non-flammable, being a non-teratogen with a minimal effect on the cardiovascular system, and having no deleterious effects on neurocognition in non-human models ^39– 41^. It was approved for adult use in the EU in 2007, and the minimum alveolar concentration of xenon in adults is 63% ^42^. The future role of xenon as a neuroprotective agent remains to be determined ^43^. A recent meta-analysis indicates that, in adults, xenon offers advantages of greater hemodynamic stability and a faster emergence from both inhalational and propofol anesthesia ^44^. A study is currently underway to examine the role of xenon as an adjuvant to inhalational anesthesia in the pediatric population undergoing interventional procedures of cardiac catheterization ^45^. The future implications for the application of xenon for sedation have yet to be explored.

## New innovations in drug delivery

### Target-controlled infusion

Innovations in computer technology, pharmacokinetic modeling, and IV infusion delivery devices have fostered the development of TCIs. TCI represents an innovative method to deliver IV drugs via computer models, with goals of achieving a defined (“target”) drug concentration at a specific body compartment or organ (brain) ^46^. TCI is used to administer propofol and opioids for sedation and general anesthesia to millions of patients every year. TCI was first described in the 1980s for clinical pharmacology research ^47^. The first-generation delivery pumps designed to specifically administer propofol were first approved in 1996, and the second generation provided the user with the ability to administer a selection of drugs (e.g. propofol and remifentanil) using different pharmacokinetic models.

Every anesthetic agent accumulates in the tissue during drug delivery. This accumulation confounds the relationship between the infusion rate set by the provider and the drug concentration in the patient. TCI devices are designed to account for the accumulation of drug in tissues and to adjust the infusion rate in order to achieve and maintain a steady-state drug concentration in the plasma or effect site ^48, 49^. When using pharmacokinetic-derived models specific to a particular drug, a TCI system incorporates patient characteristics (weight, height, age, sex, and additional biomarkers) to achieve a targeted serum-level concentration ^49, 50^. This technology constantly estimates the concentration of a drug at the target and in the plasma, allowing the clinician to make changes based on clinical or physiological (bispectral index monitoring) indicators.

The TCI systems are identified as open- and closed-loop systems. In the open-loop systems, the providers select a specific drug and a specific pharmacokinetic or pharmacodynamic model from the drug library incorporated in the device ^49^. Published models have been embedded in the pumps for propofol, remifentanil, sufentanil, and alfentanil ^46^. A limitation of this delivery technique is that it lacks real-time feedback from the patient to the TCI delivery system. Thus, continuous clinical assessment of the patient and refining of the target is often required.

**Figure 2.**
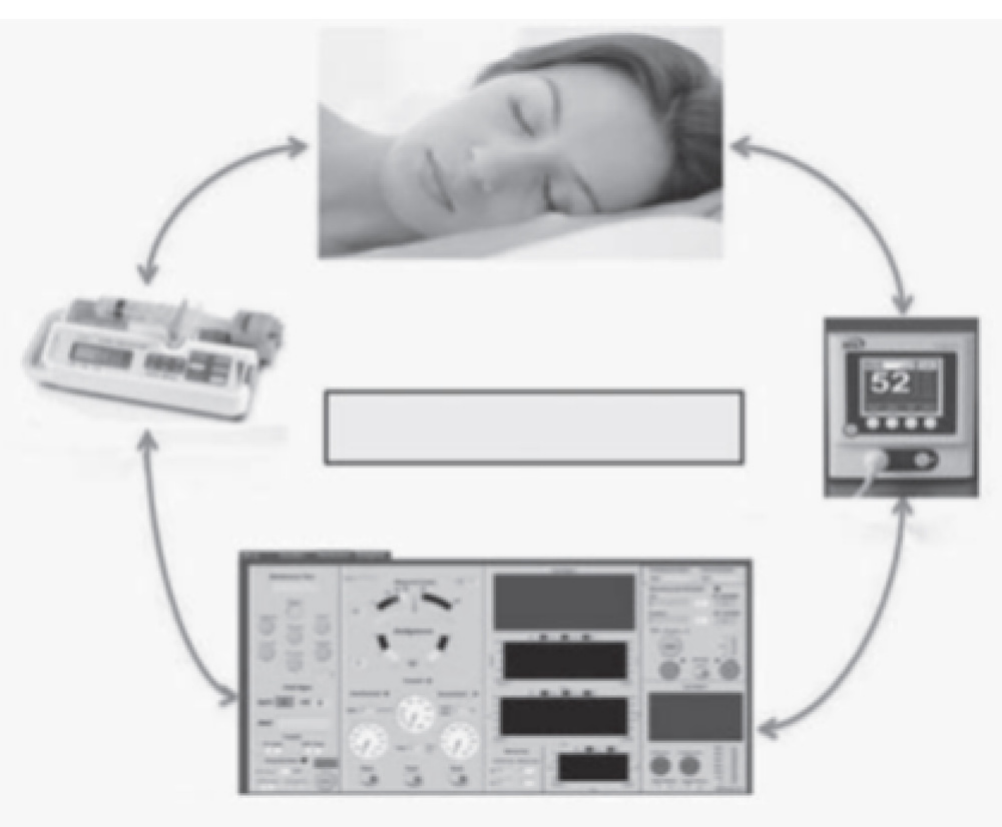
Closed-loop system for hypnosis. There are three elements in every closed-loop system: a central operating software (brain of the system), one or more target-control variables (hypnosis monitor), and one or more drug delivery systems (infusion pump). Permission was obtained for the reproduction of this figure.

A closed-loop system is a system wherein the measured output(s) is used by a controller to determine a new input to the system ( Figure 2). The controller which closes the loop can be manual or automated. A single closed-loop controller in anesthesia has been used for hypnosis ^51^, neuromuscular blockade ^52^, analgesia ^51^, arterial blood pressure control ^53^, and fluid optimization ^54^.

Currently, most TCI technology is based on plasma concentrations rather than effect-site concentrations. A recent pharmacokinetic–pharmacodynamic model used effect-site rather than plasma concentration to demonstrate that the rousability associated with dexmedetomidine reflects a response to repeated auditory stimulation ^55^. In comparison to the open-loop system, the closed-loop system may offer the advantages of more precise dosing, decrease in some workload functions, improved and standardized control of the depth of sedation and anesthesia, decreased consumption of drug, improved hemodynamic stability, faster postoperative recovery, and minimized individual operator variability in titration of the sedative agent ^56^.

## Failure of new innovations

Some technological innovations initially offered promise. However, as with any new innovation, some devices and drugs disappointed. The lack of success of these agents and devices could be related to safety, quality of anesthesia delivery, or financial reasons. In this section, we will discuss the recent failure of Sedasys, the computer-assisted personalized sedation system (CAPS), and fospropofol, both of which designed to improve the delivery and side effect profile of propofol.

### Computer-assisted personalized sedation system

Initially, there was optimism that Sedasys could revolutionize the field of non-operating room sedation by integrating continuous physiologic monitoring with patient feedback (response to auditory stimuli) to control the depth of sedation. The physiologic monitoring includes non-invasive arterial blood pressure, pulse oximetry, capnography, and electrocardiogram, all consistent with the American Society of Anesthesiologists (ASA) Practice Guidelines for Sedation and Analgesia by Non-Anesthesiologists and ASA Standards for Basic Anesthetic Monitoring ^57, 58^. This closed-loop system was intended to maintain sedation and minimize the risks of respiratory depression, cardiovascular instability, and loss of responsiveness. The first-to-market product (FDA approval 2013) in the CAPS category is the SEDASYS® Computer-Assisted Personalized Sedation System (SEDASYS® System, Ethicon Endo-Surgery, Johnson and Johnson). This technology was intended to administer mild to moderate propofol sedation only to healthy adult patients of ASA 1 and 2 for gastrointestinal (GI) endoscopic procedures.

Although SEDASYS provided a higher degree of patient and clinician satisfaction and fewer adverse events compared to any other standard-of-care delivery regimens (fentanyl and versed) ^59^, Ethicon, Inc., announced in March 2016 that it would no longer market Sedasys. Sedasys failed to gain popularity and adoption for the following reasons: 1) the device was programmed to only decrease the sedation depth (propofol dose) but not to increase it, 2) the dosing schedule was not efficient for the fast pace and turnover of most diagnostic upper GI endoscopic procedures, and 3) the device failed to satisfy both patients and providers, limited by the FDA approval for no more than moderate sedation ^60^.

Although future CAPS devices may aim for deep sedation, clinical observation skills must be utilized in conjunction with continuously and vigilantly following the patient’s respiratory, cardiovascular, and neurologic status.

### Fospropofol

Fospropofol (Lusedra Eisai Inc., Woodcliff Lake, NJ, USA) is a water-soluble phosphate ester prodrug of propofol that was FDA approved in December 2008 for monitored anesthesia care. Upon administration, fospropofol is hydrolyzed by endothelial cells and completely metabolized by alkaline phosphatase to yield propofol, phosphate, and formaldehyde ^61^. Pain on injection is observed less frequently with fospropofol than with propofol. Without lipids, egg products, or preservatives, fospropofol eliminates the allergic responses, bacterial infections, and hyperlipidemic concerns associated with propofol. Fospropofol was recently discontinued in the USA; the likely reasons include 1) its delayed onset of effect (peak effect is in 8 minutes with clinical effect in 4–13 minutes) ^62^ and slower recovery compared to propofol and 2) its significant associated perineal paresthesia and pruritus.

## Conclusion

New innovations in drug development and delivery contribute to continued improvement in anesthesia. Large clinical trials are needed to provide the greatest level of evidence and safety prior to widespread clinical implementation.
